# Structural Stability, Transitions, and Interactions within SoxYZCD-Thiosulphate from* Sulfurimonas denitrificans*: An* In Silico* Molecular Outlook for Maintaining Environmental Sulphur Cycle

**DOI:** 10.1155/2016/8683713

**Published:** 2016-09-29

**Authors:** Sujay Ray, Arundhati Banerjee

**Affiliations:** ^1^Department of Biochemistry and Biophysics, University of Kalyani, Kalyani, Nadia, India; ^2^Department of Biotechnology, National Institute of Technology, Mahatma Gandhi Avenue, Durgapur, West Bengal, India

## Abstract

Thiosulphate oxidation (an essential mechanism) serves to maintain the global sulphur cycle. Earlier experimental and computational studies dealt with environmental thiosulphate oxidation but none dealt with thiosulphate oxidation from deep ocean belts. Wet-laboratory experimental research shows that epsilon-proteobacteria* Sulfurimonas denitrificans* possess* sox* (sulphur-oxidizing) operon and perform thiosulphate oxidation efficiently underneath the oceans. From this specific* sox *operon, SoxCD complex recycles the thiosulphate-bound SoxY from SoxYZ complex to balance the environmental sulphur cycle. So, four chief proteins were variedly modeled and relevant simulated interactive structures were obtained. The final simulated tetraprotein complex (SoxYZCD) from docked SoxYZ and SoxCD complexes was disclosed to be a highly interactive one with predominant ionic residues. Free energy of folding, solvent accessibility, and conformational shifts (coil-like conformation to helices and sheets) were observed in SoxYZ complex after interacting with SoxCD. The stability of the complex (SoxYZCD) after simulation was also observed through the electrostatic surface potential values. These evaluations were rationalized via biostatistics. This aids SoxCD for recycling SoxY along with thiosulphate, which remains interconnected by four H-bonds with SoxY. Therefore, this novel exploration is endowed with the detailed molecular viewpoint for maintaining the sulphur cycle (globally) including the ocean belts.

## 1. Introduction

In the present world, the prime requisite is to maintain global sulphur balance in the environment including Wadden Sea and other associated ocean belts. This would lead to the reduction in pollution due to harmful sulphide. This therefore would make a better and safe environment to dwell in. In the foremost stage, formation of thiosulphate (S_2_O_3_^2−^) occurs from sulphide (S^2−^) in aerobic situations [1]. At this step, the end product is such that sulphur has “+2” oxidation state but it needs to be transformed to its elemental form (S^0^) [1]. In this phase, microorganisms play a crucial role to completely oxidize thiosulphate (the most abundant sulphur anion) to maintain the proper sulphur cycle throughout the environment [1]. Therefore, several paramount bioinorganic reactions are undergone by sulphur compounds. Moreover, deep ocean bed surviving microorganisms use sulphides and such varied sulphur compounds for their chemolithoautotrophy [2, 3]. One such microorganism is an epsilon-proteobacterium* Sulfurimonas denitrificans* [2, 3].

Two different groups can be categorized for the microbial metabolic reactions of sulphur anions. Either they possess the “*sox* multienzyme complex” within their genes coding for the necessary proteins or few of them follow the pathway for “reverse sulphate reduction” [4]. The “*sox* multienzyme complex organization” encodes a cluster of genes (*sox* operon, sulphur oxidizing operon) and is the most widely predominant one [4–7].

Nevertheless, it is worth mentioning that the epsilon-proteobacteria* Sulfurimonas denitrificans* (*S. denitrificans*) retain a* sox* multienzyme complex. This specific* sox* multienzyme complex consists of* soxCD *and* soxXYZAB* [8]. Intriguingly, the explicit participation of SoxYZ complex, SoxXA complex, SoxCD complex, and the SoxB protein entirely accomplishes the thiosulphate oxidation mechanism [6, 9]. Amongst them, the molybdenum-cofactor preserving protein is SoxC, while SoxD protein is a cytochrome protein of type C. It comprises duo-heme clusters: one is well known as D_1_ and the other one is known as D_2_ [10]. Altogether, SoxCD aids in the performance of sulfane dehydrogenase [10]. First and foremost, it is essential to study the interactive complex of SoxCD protein from* S. denitrificans*. Further after that, the interactive nature amongst SoxY and SoxZ proteins is necessary to be explored. After that, the SoxYZCD complex formation and the residual participation for thiosulphate oxidation are highly paramount to be examined. This is, moreover, based upon the wet-laboratory experiments and their documentation, which states SoxYZ to be crucial and to serve as an important interacting partner for SoxCD to complete the thiosulphate oxidation process [8, 9]. In the presence of SoxYZCD,* sox *system is proficient to oxidize elemental sulphur, sulphide, and explicitly sulfane sulphur (-S-) in thiosulphate [4, 6, 7, 9]. Within the complex, SoxY solely participates with thiosulphate. Further SoxCD aids in recycling the thiosulphate-bound protein SoxY from SoxYZ complex via releasing of the sulphate group from SoxY protein, hydrolytically [4, 6, 7, 9].

Formerly, the modeling of SoxC and SoxD protein from* Sulfurimonas denitrificans* was performed via simulation. A comparable study was examined for the pre- and postsimulated complexes [4]. But hitherto, there was no such molecular and residual disclosure for the analysis of the entire pattern of interaction in SoxYZCD with thiosulphate for the oxidation of thiosulphate in Wadden Sea and deep ocean belts. In addition to that, even previously the analysis of the conformational switches in SoxYZ that benefits the recycling process along with the thermodynamic stability in SoxYZ for the fulfilment of the entire phenomena in Wadden Sea and associated ocean belts was not dealt with. So, the 3D protein structures of SoxC and SoxD proteins from* S. denitrificans *were developed through comparative modeling technique. Additionally, SoxY and SoxZ proteins from* S. denitrificans *were modeled efficiently using fold recognition technique. Satisfaction of its stereochemical properties was executed, in either of the cases. The individual modeled proteins were docked amongst themselves to obtain SoxYZ and SoxCD complex, first. The models were individually optimized and simulated for a stable interactive investigation. Furthermore, the simulated SoxCD protein of* S. denitrificans *was picked to dock with the simulated SoxYZ protein from the same microorganism. Moreover, to the final SoxYZ complex, thiosulphate was docked via protein-ligand docking procedure. The detailed interactive residues indulged in the interaction of the quadracomplex; SoxYZCD was further studied. The interaction between thiosulphate and the SoxYZ complex was also analyzed. The stability and conformational alterations in SoxYZ protein upon SoxCD interaction for its recycling were additionally examined. All deductions were substantiated with relevant statistical significance.

Therefore, this novel present scenario provides a way out to examine the fields of binding, stability, conformational switches, and the arrangement of interactions in SoxYZCD protein from* S. denitrificans* (epsilon-proteobacteria). It also examines the involvement of thiosulphate for getting recycled by binding to SoxY. This might cover up a massive part for thiosulphate oxidation in dark ocean beds for the chemolithoautotrophy of microorganisms.

## 2. Methodology

### 2.1. Analysis of the Respective Amino Acid Sequences and Template Exploration for Homology Modeling of SoxC and SoxD from* Sulfurimonas denitrificans*

In our previous investigation, we analyzed the residual interaction pattern between two essential proteins, SoxC and SoxD, from* Sulfurimonas denitrificans* for global sulphur oxidation [4]. For that purpose, we modeled the 3D structures of SoxC and SoxD protein and further disclosed the residue-level binding strength between two modeled proteins [4]. The comparative study was examined for the stability of SoxC and SoxD protein after the duo-protein was simulated. In this present study, first we have opted for an improved process for the preparation of the homology models of the two paramount proteins.

The individual amino acid sequences of SoxC and SoxD from* S. denitrificans* were obtained from UniProtKB (accession numbers Q30NU7 and Q30NU8 for SoxC and SoxD, resp.). The sequences were substantiated from NCBI protein sequence database too [4]. For template selection, templates were obtained by SWISS-MODEL template searching procedure. With the usage of varied force fields, the explorations of templates for the individual proteins (SoxC and SoxD) were performed [11]. The same templates were also obtained by the utilization of PSI-BLAST with reference to PDB. PSI-BLAST helps to bring the distantly related species into consideration and is also acknowledged to be more sensitive when compared to PBLAST [12].

The respective templates for building the respective comparative (homology) models were from the X-ray crystal structure of

(i) *Paracoccus pantotrophus* for SoxC (PDB code: 2XTS_A with 87.00% query coverage sharing 43.78% sequence identity,* E*-value: 2*e* − 114),
(ii) *Paracoccus pantotrophus* for SoxD (PDB code: 2XTS_B with 51.00% query coverage and sharing 37.82% sequence identity,* E*-value: 8*e* − 38).

### 2.2. Molecular Modeling of the Four Respective Proteins

#### 2.2.1. Homology Modeling for SoxC and SoxD Proteins from* Sulfurimonas denitrificans*

SoxC and SoxD protein models were constructed utilizing homology modeling with the aid of Swiss Model software [11]. The modeled proteins were selected from the GMQE (Global Model Quality Estimation) score. The range for GMQE score is from zero to one. It infers the accuracy of the protein model with higher values implying higher dependability [11]. Furthermore, the next step for preferring the best modeled protein is to examine the sequence identity of the target and template sequence. The GMQE values are 0.70 and 0.38 for SoxC and SoxD, respectively. Additionally, the QMEAN score was also supportive with −3.56 and −3.45 for SoxC and SoxD, respectively.

The individual homology models were superimposed upon their relevant crystal templates to observe the root mean square deviations (RMSD) with the aid of PyMOL. DaliLite Server (Analysis Tool Web Services from the EMBL-EBI, 2013) from EMBL packages also helped to validate the results. 0.068 Å and 0.079 Å were the RMSD values with respect to C_*α*_ atoms for SoxC and SoxD, respectively. This implies that the models were potentially akin to their crystal template structures [13].

#### 2.2.2. Modeling via Fold Recognition/Threading for SoxY and SoxZ Proteins from* Sulfurimonas denitrificans*

For the purpose of modeling the SoxY and SoxZ proteins, fold recognition technique was utilized by operating Phyre2, rather than utilizing traditional homology modeling technique [14]. To overcome the certain specific problems, fold recognition technique (threading) was utilized for this novel study.

Fold recognition is a technique for modeling a specific protein that demonstrates the comparable fold with respect to the proteins of well-known tertiary structures. So, Phyre2 was operated here for the execution of fold recognition [14]. In this method, first, the query sequence is used to perform PSI-BLAST to classify remotely related amino acid sequences [12]. The usage of hidden Markov model (HMM) was to produce a familiar sequence profile. This profile is utilized to explore against a HMM database of well acknowledged structure [14]. Template ID from fold library, chain, PDB ID, confidence score, and query coverage were* c4uwqK, *chain K, 4UWQ_K, 100%, and 77%, respectively, for SoxY protein. Template ID from fold library, chain, PDB ID, confidence score, and coverage were* d1v8ha1*, chain A, 1V8H_A, 100%, and 99%, respectively, for SoxZ protein.

### 2.3. Optimization of the Distorted Loop Regions for Conformational Stability

During homology modeling, when the sequence identity between the protein to be modeled and its competent template remains within the range of 30% to 50%, then certain disparities in loop conformations and fallacies might occur in the side-chain packing [15–17]. This often leads to instability in the protein [16, 17]. Such loop regions normally reside in the disallowed regions of the Ramachandran plot. So, optimization and remodeling of the loop regions are essential to accomplish the proper orientation of *ψ*-*φ* angles. This, therefore, eradicates such deformities. ModLoop via MODELLER was operated for the establishment of the loop optimization [18].

### 2.4. Refinement and Energy Optimization of the Individual Modeled Protein Structures

For the refinement and energy optimization of the single loop optimized protein structures (“SoxY, SoxZ, SoxC, and SoxZ”), an efficient atomic-level algorithm, ModRefiner, was followed [19]. The algorithm executes via a combination of two different force fields: physics-based and knowledge-based. In terms of the proper H-bonding, topology of backbone, and positioning of side chains, the proteins were well optimized by drawing them closer to their native states, where the highest state of interaction exists [19].

### 2.5. Substantiation of the Stereochemical Properties of the Modeled Structures

The stereochemical properties of the four homology modeled structures were substantiated by SAVES server. To analyze the residue-level profile of individual amino acid from the respective protein 3D models, VERIFY3D was examined [20]. Examination of Ramachandran plots from PROCHECK [21] web server revealed that most of the residues occupied the most preferred regions with zilch amino acids in the disallowed regions [21, 22].

### 2.6. Molecular Docking Studies of SoxY, SoxZ, SoxC, and SoxD from* Sulfurimonas denitrificans*

First, SoxYZ and SoxCD complexes were formed which was followed by the docking of the duo-protein complexes amongst themselves to form the SoxYZCD complex. For this purpose, at each case, the entirely automated program ClusPro 2.0 was operated [23, 24]. For the individual duo-complexes (SoxYZ and SoxCD), both SoxY and SoxC, being longer, served to be the receptors while the other two modeled proteins were uploaded as ligands. But for docking the duo-complexes, SoxCD served as receptor and SoxYZ as ligand. The unstructured residues were confiscated by the execution of the advanced option of ClusPro 2.0. In each of the cases, amongst all the 10 docked structures, the finest cluster sized model was opted for for further investigations. Docking outcomes from ZDOCK and GRAMM-X were shown to be inclusive of the results [25, 26].

### 2.7. Energy Minimization of Docked Protein Complexes

The modeled and docked complexes were energy optimized using CHARMM force field to diminish the net overall energy. It was also performed to alter the respective conformations of the dihedral angles to allow them to vary concurrently [27]. The steepest descent technique preceded by conjugate gradient mechanism was executed through operation of Discovery Studio software [27]. The phenomena continued till the modeled structures individually attained a final RMS gradient of 0.0001 [27].

### 2.8. Molecular Dynamics Simulation of the Docked Complexes

To attain a stable conformation for the individual protein complexes with an overall optimization of the net energy, two-step Molecular Dynamics (MD) simulation was performed for the betterment of the respective interactions. Initially, by template information of modeled structures and based on the principle of MD simulation, Fragment-Guided Molecular Dynamics (FG-MD) was operated [28]. It first disassembles (fragments) the provided docked protein structure and further assembles each fragment to accomplish the energy optimized protein modeled structure [28]. The physical based potentials, except for AMBER99, explicitly carried the decoys away from the native protein state [28]. FG-MD was extensively tested with experimentally validated X-ray crystal structures and demonstrated noteworthy potential in the protein structure refinements at the atomic level. It was observed to satisfy the entire conformational requirements. The complete simulation process comprises mainly three steps: (i) identification and analysis of the fragment structure, (ii) utilization of the simulated annealing technique for MD simulation, and (iii) selection of the final model [28]. The steric clashes were removed accompanied by the upgradation of the torsion angle. Thus, every time the structure is brought closer to its native state with a higher standard of accuracy [28].

Further, these reconstructed optimized complexes from its fragments were simulated via the operation of the Chiron Energy Minimization tool [29]. CHARMM force field is pursued for the optimization of the whole docked complex at a time [27]. Further, a high temperature exchange rate for the complexes is utilized with subjection to simulations by Molecular Dynamics (MD) [29]. Thus, finally, not only were the steric clashes purged rapidly but also minimal disconcertion of the backbone of the protein was achieved [29].

### 2.9. Analysis of the Interactive Residues and Binding Patterns in SoxYZCD Tetracomplex

With the aid of Protein Interaction Calculator (PIC), several interactions like disulphide bonds, main-chain side-chain interactions, and hydrophobic, ionic, aromatic-aromatic, aromatic-sulphur, and cation-*π* interactions were examined [30]. Analysis of the interactive docked complexes was also carried out using the Analyze Binding Site tool from Discovery Studio (DS) package. The result served to be analogous to the outcomes from PIC. The main focus was to examine the ionic interactions, if any. This is because the ionic interaction predominantly plays a pivotal role in strengthening the interaction [31].

### 2.10. Stability Evaluation of the SoxYZ Complex upon SoxCD Interaction

#### 2.10.1. Estimation of Free Energy of Folding

For the examination of the stability of SoxYZ complex after participation of SoxCD complex (for the recycling of SoxYZ), free energy of folding for SoxYZ complex was assessed and compared (before and after the interaction of SoxCD complex). It was evaluated with the support of VADAR 2.0 [32]. A descent in the free energy of folding implies better folds and a strong interactive protein complex.

#### 2.10.2. Conformational Switches in SoxYZ Complex upon SoxCD Interaction

Increment in the percentage of residues adopting turn-like and helical conformations led to stronger interaction with a better conformational stability in the protein [33, 34]. Furthermore, ascent in the residues involved in turn-like regions leads to firmer backbone flexibility which additionally aids the protein to interact with its partner proteins. Moreover, it has been previously investigated with evidence that proteins exhibiting few 3_10_ helices accompanied by the increased percentage for pure *α*-helices are conformationally steadier than the proteins adopting only pure *α*-helices [35]. So, the secondary structure distribution in the SoxYZ protein complex was evaluated and compared utilizing DSSP method, before and after its interaction with SoxCD protein complex [36]. Discovery Studio packages of Accelrys and PyMOL also authenticated the results [13]. The stability and strength of interaction of the SoxYZ complex were thus again perceived through the conformational switches.

#### 2.10.3. Estimation of the Net Area for Solvent Accessibility in SoxYZ Complex before and after Interaction with SoxCD Complex

Calculation, analysis, and comparison of the solvent accessible surface area for the essential interacting amino acids residues from SoxYZ protein complex were performed. The specific amino acid residues that take part in the interaction pattern were taken into consideration from the preinteractive complex of SoxYZ with SoxCD and after the tetracomplex formation. It was observed to rationalize the aforementioned energy estimation outcomes [37]. As the interaction grows stronger, the area available for solvent will keep descending [37].

### 2.11. Estimation of the Electrostatic Potential on the Surface of the SoxYZ Protein Complex

The literature suggests that more negative electrostatic potential value apprehends a better interaction of the protein complex of interest and thereby leads to an overall complex stability [38]. The pictorial representation of the electrostatic mapping upon the protein surface was illustrated by the assistance of PyMOL (MOLecular viewer using Python language) [13]. So, the optimized complex structure and the preoptimized tetracomplex structure were further utilized for the electrostatic potential mapping upon the surface of the protein. Electrostatic surface potential was evaluated via vacuum electrostatics in kT/e units, with the support of PyMOL [13].

### 2.12. Rationalization through Statistical Significance

To examine and rationalize all the estimated outcomes through statistical significance, *P* value was evaluated through the paired* t*-test in each of the cases. In this* t*-test, the only assumption usually accepted is that the standard deviations (SDs) are not approximately akin to both measurements for the individual cases (before and after SoxCD interaction). *P* < 0.05 affirms the results to be statistically significant.

### 2.13. Protein-Ligand Docking for SoxYZCD Complex with Thiosulphate

Analysis of the interaction amongst the essential proteins upon the involvement of the sulphur anion, thiosulphate, was also essential. Thiosulphate was obtained from PubChem and was docked as a ligand with the receptor (final stable SoxYZCD protein complex). AutoDock Vina was operated for the purpose [39]. Not only the condition and declaration of the search space but also the specification of the binding region was provided. Further again, the molecular surface of the protein-ligand complex was computed with the utilization of segmentation algorithm using PatchDock [40]. Analogous outcomes for the docked protein-ligand complex were obtained. Further, the best suited and finest model was opted for, depending upon the relevant data for interaction energy, that is, Gibbs free energy (Δ*G*). The SoxYZCD-thiosulphate model having the maximum and best suited (−) Δ*G* value is chosen for having the highest strong binding affinity.

### 2.14. Analysis of the Protein-Ligand Interactions and Binding Patterns in the Final SoxYZCD-Thiosulphate Complex

The obtained final complex of SoxYZCD-thiosulphate was analyzed with the aid of PyMOL plugins and the interacting residues and binding patterns were thereby examined [13]. Thus, the complete model having four interactive proteins and a ligand (thiosulphate) was investigated with the establishment of the interactive residues for the purpose of recycling the thiosulphate-bound SoxY protein from the SoxYZ complex with the aid of SoxCD.

## 3. Results and Discussion

### 3.1. Portrayal of the 3D Functional Structure of the SoxC Protein

**Figure 1:**
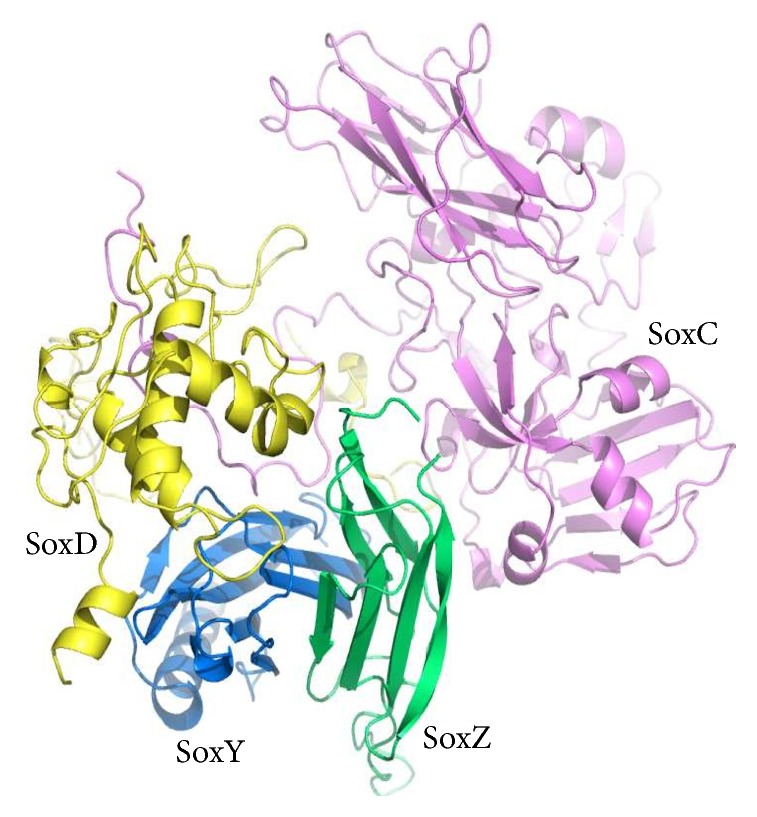
Pictorial representation of the SoxYZCD complex from* Sulfurimonas denitrificans* with magenta shaded SoxC, yellow shaded SoxD, blue shaded SoxY, and green shaded SoxZ.

The tertiary structure for the homology modeled SoxC protein from* Sulfurimonas denitrificans* was analogous to its template from* Paracoccus pantotrophus* (PDB code: 2XTS; A chain). The 391-residue-long protein comprises mainly 18 sets of *β*-sheets and 10 sets of *α*-helices interspersed with coil regions. 14.3% of the amino acid residues contributed to the respective 10 *α*-helices, whereas 29.2% of the residues were responsible for the adoption of *β*-sheet conformation in the protein. The modeled SoxC structure is well illustrated in Figure 1 with red, cyan, and magenta shades representing *β*-sheets, *α*-helices, and interspersing coils, respectively.

### 3.2. Portrayal of the 3D Functional Structure of the SoxD Protein

The tertiary structure for the homology modeled SoxD protein from* Sulfurimonas denitrificans* was also analogous to its template from* Paracoccus pantotrophus* (PDB code: 2XTS; B chain). The homology modeled SoxD 3D structure was 216 residues long. Only two sets of antiparallel *β*-sheets were observed in the 3D structure with the involvement of 2.8% of the amino acid residues. 25.5% of the amino acid residues participated in the configuration of *α*-helices. The entire protein was interspersed with coil regions. SoxD structure is illustrated in Figure 1 with *α*-helices and coils in yellow and pink shades, respectively.

### 3.3. Portrayal of the 3D Functional Structure of the SoxY Protein

The functional tertiary structure for the homology modeled SoxY protein from* Sulfurimonas denitrificans* was 122 amino acids long. SoxY protein comprises mainly 8 sets of *β*-sheets and 2 sets of *α*-helix conformations at the N-terminal end (residues: 2–20 and 59–61). They were observed to remain interspersed with turn-like regions. The homology modeled protein begins with Asp1 forming a small turn. The model is well presented in the interactive picture of Figure 1 with *α*-helix, *β*-sheets, and turn-like regions in marine-blue shade.

### 3.4. Portrayal of the 3D Functional Structure of the SoxZ Protein

The functional tertiary structure for the homology modeled SoxZ protein from* Sulfurimonas denitrificans* was 97 amino acid residues long. SoxZ protein comprises mainly 8 sets of parallel and antiparallel *β*-sheets linked with *β*-turns. The *β*-sheets occupy 47.82% of the entire structure. The model is well presented in the interactive picture of Figure 1 with *β*-sheets and *β*-turns in lime-green shade.

### 3.5. Analysis of Interactive Residues and Binding Patterns in the SoxYZCD Complex

**Figure 2:**
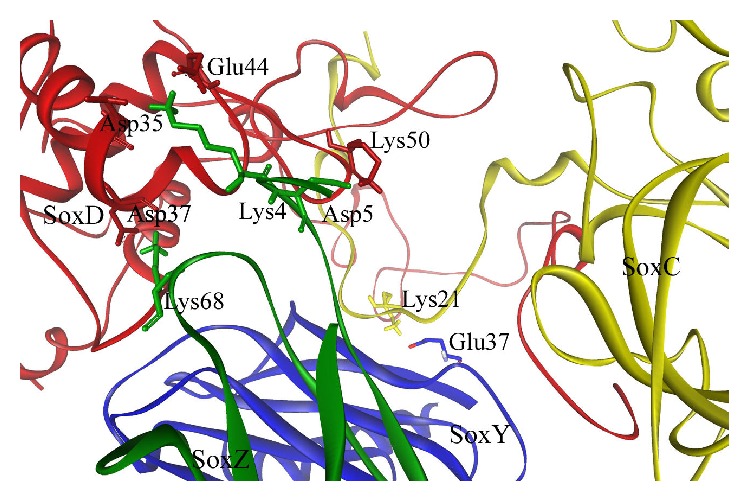
Predominant ionic-ionic interactions in SoxYZCD complex with few responsible residues labeled.

The exploration of the interactive residues with the binding patterns of the SoxYZCD tetracomplex using PIC web server and Discovery Studio Platform from Accelrys software displays the four proteins to participate strongly via 9 sets of ionic interactions. Several H-bonding interactions were perceived indulging the main chains and the side chains of the four individual proteins.  Table 1 tabulates the ionic interactions in the optimized and simulated tetracomplex (SoxYZCD) with a pictorial representation through Figure 2, showing few paramount residues for the firmer interaction.

The polar negatively charged Asp2 and Glu31 from SoxC protein participated strongly in the interaction pattern. Polar positively charged Lys21 interacted well for recycling SoxY. From SoxY, again the polar positively charged Lys34 and Arg93 were observed to actively participate in the formation of two ionic interactions with Glu8 and Asp57 from SoxZ. Lys4, a polar positively charged residue from SoxZ, further formed two ionic interactions with Glu44 and Asp35 from the SoxD protein. This was additionally supported by the interaction of polar negatively charged Asp5 (adjacently positioned to Lys4 that formed two ionic interactions) from SoxZ with Lys50 from SoxD for a better stability to the tetracomplex. Furthermore, Lys61, Arg67, and Asp37 from SoxD participated strongly in building up a strong electrostatic interaction in the SoxYZCD tetracomplex. Altogether, these interactions helped to accommodate the partner proteins to interact firmly.

### 3.6. Estimation from the Stability Parameters before and after Interaction with SoxCD

#### 3.6.1. Free Energy of Folding

To analyze and compare the stability of SoxYZ complex after interaction with SoxCD complex, free energy of folding was estimated. VADAR 2.0 [32] aided to disclose a decline in the value from −144.71 kcal/mol (in SoxYZ complex) to −577.31 kcal/mol for the SoxYZCD complex (Table 2). Therefore, the reduction in the free energy of folding indicates spontaneous interaction with better folds in the SoxYZ protein complex after interaction with SoxCD complex.

#### 3.6.2. Structural Transitions in SoxYZ upon SoxCD Interaction

**Figure 3:**
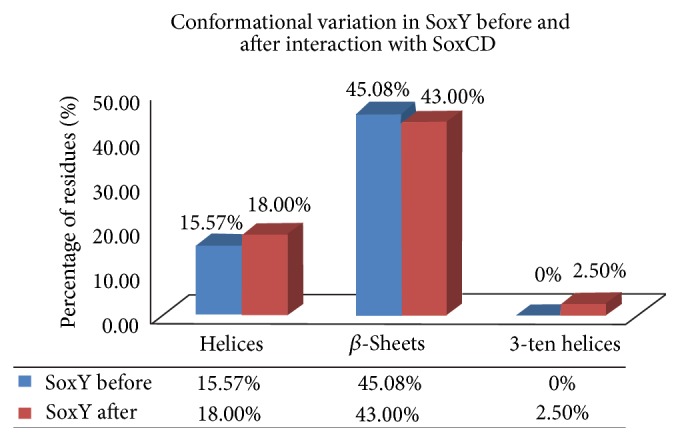
Comparative analysis for the transitions in the conformation of SoxY upon SoxCD interaction.

On comparison and analysis of the conformational switches in the SoxYZ complex before and after interaction with SoxCD complex, it was disclosed that both SoxY and SoxZ underwent abrupt conformational alterations upon the participation of SoxCD in recycling SoxY protein. For SoxY, there was an abrupt increase in helical regions from 15.57% to 18%. Thereby, it leads to a firmer conformation. Captivatingly, the presence of few 3_10_ helices in SoxY protein (after SoxCD interaction) accompanied by the increased percentage of residues adopting pure *α*-helices additionally led to a firmer and steadier conformation in the protein (Figure 3) [35]. The increase in percentage of pure *α*-helices with the presence of 3_10_ helices and *β*-sheet conformations in the SoxY protein (after SoxCD interaction) makes SoxY exhibit a steadier and more interactive conformation [34, 35].

**Figure 4:**
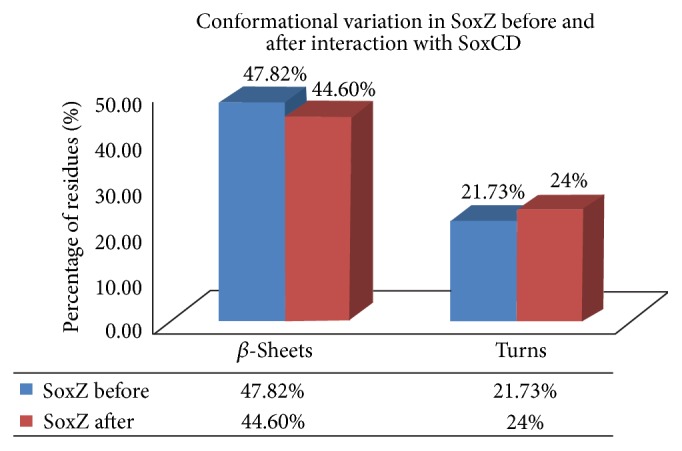
Comparative analysis for the transitions in the conformation of SoxZ upon SoxCD interaction.

**Figure 5:**
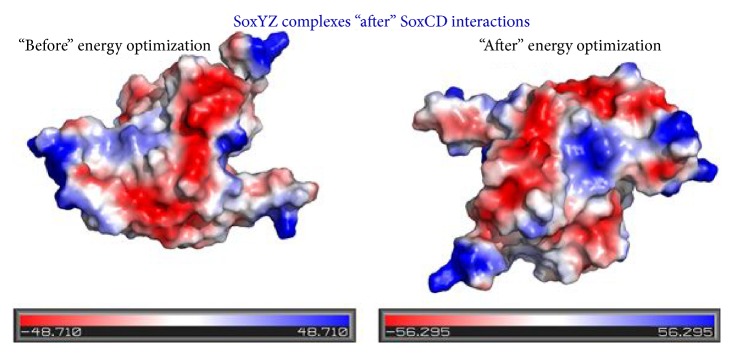
Comparative analysis for the fluctuation in the electrostatic potential on the surface SoxYZ protein complex (before and after optimization) upon SoxCD interaction.

Again, the increment in the *β*-turn conformations in SoxZ from 21.73% to 24.00% after SoxCD interaction (Figure 4) affirms even SoxZ to exhibit a stronger conformation and better stability [33]. Thereby, the entire conformational shift in SoxYZ protein complex benefits the recycling process of SoxY protein by SoxCD. The pictorial representation with tabulation of the conformational switches is illustrated in Figures 4 and 5, respectively, for SoxY and SoxZ proteins.

#### 3.6.3. Net Area Available for Solvent Accessibility

This helped to affirm that SoxYZCD complex interacted strongly. Thus, it gets aided to be easily pulled for recycling. Herein, from Table 2, the net area available for solvent accessibility from the interacting residues in SoxYZ duo-protein complex in SoxYZCD complex was perceived to get decreased from 486.92 Å^2^ to 153.92 Å^2^, after interaction. There was even a decline in the overall value for net area of solvent accessibility (considering the summation of the accessible area values from all the residues from SoxY and SoxZ) from SoxYZ protein.

### 3.7. Electrostatic Potential on the Protein Surface of SoxYZ from SoxYZCD Entire Complex before and after Optimization Followed by Simulation

In kT/e units, electrostatic surface potential displayed the postoptimized and simulated SoxYZCD protein complex to exhibit better and firmer interaction (Figure 5). The blue region symbolizes the electropositive regions, while the red shaded ones are for the electronegative areas. A vivid illustration from Figure 5 provides the notion that, from the tetraprotein complex, SoxYZ complex (after optimization and simulation) exhibited an enhanced stability. The alteration in the electrostatic potential on the SoxYZ surface was from ±48.710 to ±56.295. Therefore, the optimization and simulation further affirm rendering a more stable interaction [38].

### 3.8. Rationalization of the Data Evaluated via Statistical Significance

The respective outcomes for the alterations in SoxYZ complex upon SoxCD interaction were observed to be statistically significant with an evaluated *P* value of less than 5% in each of the calculations. The *P* values for the alterations in the free energy of folding and net area for solvent accessibility values were 0.021342 and 0.01218, respectively. Furthermore, again the conformational switches in SoxY were observed to be statistically significant with a *P* value of 0.011327 and 0.012138 for the ascent in the helical conformations and for the decrease in the *β*-sheet conformations (to allow few residues to adopt 3_10_ helices). *P* value of 0.021231 for the presence in the residues undergoing 3_10_ helices, which accompanied the pure *α*-helical conformations, also is statistically significant. Additionally, even the conformational shifts in SoxZ (having no helices) were also perceived to be statistically significant with a *P* value of 0.013219 and 0.005136 for decrement in *β*-sheets (to allow few residues to exhibit an increase in the turn-like regions) and increment in turn-like regions, respectively.

### 3.9. Interacting Residues and Binding Patterns for the SoxYZCD-Thiosulphate Complex

**Figure 6:**
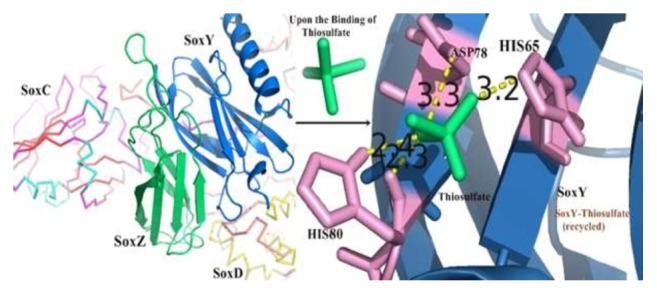
Illustration for the entire mechanism for interaction of SoxYZCD complex with thiosulphate compound and thereby recycling of SoxY-thiosulphate complex (on right). The interactions between thiosulphate and SoxY are shown in yellow dashes.

Along with the strong and stable ionic interactions within the tetraprotein complex (SoxYZCD), thiosulphate binding interactions were therefore analyzed. For the interaction between SoxY and thiosulphate (to get recycled), mainly His65, His80, and Asp78 from SoxY formed H-bonds as depicted in Figure 6. Strong hydrogen bonds were accomplished by these aforementioned residues from SoxY protein with the thiosulphate molecule. Formation of a cavity in SoxY by the positively charged environment of SoxZ helps to accommodate the thiosulphate ligand within the protein strongly. This thereby leads to getting the thiosulphate molecule recycled along with SoxY by SoxCD.

Previously, other molecular level studies involving manifold molecular and computational probes were examined with several species of proteobacteria like* Allochromatium vinosum, P. pantotrophus, Pseudaminobacter salicylatoxidans *(KCT001), and many more [5–7, 41, 42]. In addition to that, molecular level studies were also explored for the eradication of environmental pollutants like phenol [43]. Literature study documents the species (mainly proteobacteria) to perform thiosulphate oxidation, but a computational investigation into the oxidation of sulphur after the formation of the tetracomplex protein (SoxYZCD) from* S. denitrificans* (which is responsible for performing thiosulphate oxidation on a major part on this earth including deep ocean belts) was nowhere dealt with. So this novel molecular analysis with the epsilon-proteobacteria* Sulfurimonas denitrificans* was essential to be explored. The overall study of this research work has been depicted through Suppl. Figure 1 in Supplementary Material available online at http://dx.doi.org/10.1155/2016/8683713. To summarize, the four relevant energy optimized Sox proteins, “SoxY, SoxZ, SoxC, and SoxD,” were modeled discretely after the satisfaction of their necessary stereochemical properties. Firstly, the necessary duo-complexes (SoxYZ and SoxCD) were formed which was further followed by the generation of the tetracomplex SoxYZCD. The finally docked best modeled tetracomplex of SoxYZCD was energy optimized and underwent two-step Molecular Dynamics simulation further.

To examine and explore the residual participation and the binding pattern for the steady simulated SoxYZCD complex, the predominant strengthening ionic interactions were disclosed. Several hydrogen bonding interactions also supported the interactive binding pattern. Nine sets of ionic interactions in the entire SoxYZCD complex made the complex a firmly interactive one. The polar negatively charged Asp2 and Glu31 from SoxC protein participated strongly in the interaction pattern. Several other paramount residues helped in the proper accommodation of SoxCD protein complex through pocket formation in SoxYZ complex. Furthermore, the thiosulphate molecule was observed to interact with SoxY protein efficiently with strong H-bonds by the participation of Asp78, His65, and His80 amino acids. Overall interactions between the four proteins and the thiosulphate molecule (with SoxY) aided in dragging thiosulphate-bound SoxY protein for its recycling from its respective duo-complex (SoxYZ). Thus, these residual contribution and binding patterns help in the performance of sulphur oxidation mechanism potentially. From the biological point of view, it was observed that SoxZ protein interacted strongly with more ionic interactions to participate with SoxCD complex. This joint effort therefore aided in recycling the SoxY protein, only to which the thiosulphate molecule binds, for the recycling of the SoxY-thiosulphate complex.

Several stability calculating parameters were considered with supportive statistical significance for substantiation. Evaluations from Δ*G* values in free energy of folding, net area available for solvent molecules, and electrostatic potential upon SoxYZ protein surface (an abrupt increase in the negative value) displayed that the sole SoxYZ protein turned out to be more stable and firmer after interaction with SoxCD complex. These altogether enabled SoxCD to recycle SoxYZ complex more easily. The conformational transitions in SoxYZ complex also help to analyze the improved conformational stability and steady interaction of SoxYZ complex in the presence of SoxCD. In SoxY, the statistically significant increase in the pure *α*-helices accompanied by 3-ten helices and *β*-sheets presents the structure to become a more stable and interactive one. Likewise, SoxZ (having no helical regions) also experienced conformational alterations with increased *β*-turn conformations. This led to the better backbone flexibility in the proteins permitting them to easily interact with their partner proteins. This further affirms the plausible recycling of SoxY protein from the SoxYZ complex, by SoxCD complex.

So, herein, the deep seas residing microorganism (*S. denitrificans*) was explored for the first time. The formation of SoxYZ and SoxCD and further SoxYZCD complex formation as well as their residual disclosure with importance to their respective positions were disclosed. Additionally, the conformational shifts and thermodynamic stability in SoxYZ protein complex for recycling SoxY-thiosulphate for the progression of sulphur oxidation mechanism served to analyze the core basis for rendering a toxic-free environment.

## 4. Conclusion and Future Scope

Finally, the outcomes of this present study focused mainly on investigating the conformational transitions and stability of SoxYZ complex upon the interaction of SoxCD protein complex from the epsilon-proteobacteria* S. denitrificans*. In addition to that, it finally also dealt with the interaction and cooperative participation of thiosulphate for recycling SoxY-thiosulphate complex from the SoxYZ complex, with the aid of SoxCD protein complex. The 3D homology modeled functional protein structures of SoxY, SoxZ, SoxC, and SoxD were analyzed and demonstrated, followed by optimization. The relevant necessary interactions in the optimized and simulated SoxYZCD protein complex were explored from the respective positions of the pertinent proteins. Predominantly, 9 sets of interactions for this recycling phenomenon for thiosulphate oxidation were served by the ionic interactions from mainly SoxY, SoxC, SoxD, and SoxZ. The stability and stronger conformation for SoxYZ protein upon interaction with SoxCD protein complex were explored further with corroboration of statistical significance. The SoxYZ complex was observed to become more stable after SoxCD interaction. Additionally, in the presence of SoxCD, SoxYZ protein complex also interacts firmly and steadily. This finally forms the steady interactive tetracomplex. Further, on the interaction with thiosulphate, four strong H-bonding interactions were observed to strengthen the protein-ligand interaction. From this stable simulated tetracomplex formation, SoxY-thiosulphate gets susceptible to being dragged out for getting recycled by SoxCD protein complex. Though there exist former investigations on the cooperation of SoxYZ and SoxCD proteins from other proteobacteria species for thiosulphate oxidation, a comparative* in silico* examination into the interactive pattern, binding residues, and conformational switches for the recycling of SoxY-thiosulphate to oxidize thiosulphate (from the Wadden Sea and associated ocean belts) by SoxCD complex of epsilon-proteobacteria (*S. denitrificans*) was not delved into hitherto. Thus, this present novel research presents a rational outlook to explore and analyze the SoxCD complex participation with SoxYZ protein complex from* Sulfurimonas denitrificans*, an epsilon-proteobacterium. This aids in maintaining a systemic sulphur cycle in the biota, even giving importance to the ocean belts.

The study further instigates the computational analysis of the participation of other proteins from the same microorganism for the help in thiosulphate oxidation. It is also exciting to investigate any mutational alterations that might affect the interactive pattern among the proteins and thereby might alter the sequential cellular mechanism. The computational investigation for the sustainability of the global sulphur balance through essential microorganisms can also be provoked in future.

## Supplementary Material

The overall flowhart for the entire study has been depicted in a pictorial manner. The figure begins with the interactive complex formed due to interactions of SoxY, SoxZ, SoxC and SoxD proteins which further interacts with the thiosulphate (ligand). The interacting residues within the entire protein-protein complex (SoxYZCD) were shown in the left hand side. On the right hand side, the interacting residues and the hydrogen bonds formed upon interaction with thiosulphate was also depicted. Furthermore, for the analysis of steady interaction after simulation, the electrostatic surface potentials upon SoxYZ protein complexes and conformational fluctuations in SoxY as well as SoxZ protein upon SoxCD interaction was illustrated through figures. The table at the end shows the steady and spontaneous interaction within the complex (through the calculation of statistically significant free energy of folding value and net area for solvent accessibility for the interacting residues).

## Figures and Tables

**Table 1:** Predominant ionic-ionic interactions in the stable simulated SoxYZCD complex.

Position	Residue	Protein	Position	Residue	Protein
2	ASP	C	61	LYS	D
21	LYS	C	37	GLU	Y
31	GLU	C	67	ARG	D
34	LYS	Y	8	GLU	Z
35	ASP	D	4	LYS	Z
37	ASP	D	68	LYS	Z
44	GLU	D	4	LYS	Z
50	LYS	D	5	ASP	Z
93	ARG	Y	57	ASP	Z

C, D, Y, and Z represent SoxC, SoxD, SoxY, and SoxZ proteins from the final tetraprotein complex, respectively.

**Table 2:** Comparable analysis for the stability of the SoxYZ protein complex (before and after interaction with SoxCD complex).

Stability parameters	Before interaction	After interaction
Free energy of folding (kcal/mol)	−144.71	−577.31
Net area for solvent accessibility of interacting residues	486.92 Å^2^	153.92 Å^2^

## Abbreviations

SDs: Standard deviations
PIC: Protein Interaction Calculator
DS: Discovery Studio
PyMOL: MOLecular viewer using Python language
MD: Molecular Dynamics.

## Additional Points

*Highlights*. The main highlights of this research are (i) the molecular level sulphur oxidation in epsilon-proteobacteria; (ii) molecular modeling and optimization of the four most essential proteins; (iii) molecular docking simulations for duo-protein complexes, tetracomplex, and protein-ligand complex; (iv) examination of responsible interactions, binding patterns, and amino acid residues at their respective positions; (v) calculation of stability parameters and comparative conformational switching with biostatistical analysis.
