# Corrigendum to “Structural Stability, Transitions, and Interactions within SoxYZCD-Thiosulphate from* Sulfurimonas denitrificans*: An* In Silico* Molecular Outlook for Maintaining Environmental Sulphur Cycle”

**DOI:** 10.1155/2016/7905302

**Published:** 2016-12-20

**Authors:** Sujay Ray, Arundhati Banerjee

**Affiliations:** ^1^Department of Biochemistry and Biophysics, University of Kalyani, Kalyani, Nadia, India; ^2^Department of Biotechnology, National Institute of Technology, Mahatma Gandhi Avenue, Durgapur, West Bengal, India

In the article titled “Structural Stability, Transitions, and Interactions within SoxYZCD-Thiosulphate from* Sulfurimonas denitrificans*: An* In Silico* Molecular Outlook for Maintaining Environmental Sulphur Cycle” [1], we found three sentences that need to be modified in the third section: Results and Discussion. In Section  3.1, the sentence “The modeled SoxC structure is well illustrated in Figure  1 with red, cyan, and magenta shades representing *β*-sheets, *α*-helices, and interspersing coils, respectively” should be changed to “The modeled SoxC structure is well illustrated in magenta shades in Figure  1 with prominent secondary structure conformations (*β*-sheets, *α*-helices, and interspersing coils).” In Section  3.2, the sentence “The entire protein was interspersed with coil regions. SoxD structure is illustrated in Figure  1 with *α*-helices and coils in yellow and pink shades, respectively” should be changed to “The entire protein was interspersed with coil regions. SoxD structure is illustrated in Figure  1 with yellow shades showing the prominent secondary structures possessing *α*-helices and coils.” Finally, the sentence “The pictorial representation with tabulation of the conformational switches is illustrated in Figures  4 and  5, respectively, for SoxY and SoxZ proteins” should be changed to “The pictorial representation with tabulation of the conformational switches is illustrated in Figures  3 and  4, respectively, for SoxY and SoxZ proteins” in Section  3.6.2.
