# Supplementary material for: Structural Stability, Transitions, and Interactions within SoxYZCD-Thiosulphate from Sulfurimonas denitrificans: An In Silico Molecular Outlook for Maintaining Environmental Sulphur Cycle
Source: J Biophys. 2016 Sep 29;2016:8683713. doi: 10.1155/2016/8683713 (PMC5061964; doi:10.1155/2016/8683713)
Supplement: Supplementary file 1 — The overall flowhart for the entire study has been depicted in a pictorial manner. The figure begins with the interactive complex formed due to interactions of SoxY, SoxZ, SoxC and SoxD proteins which further interacts with the thiosulphate (ligand). The interacting residues within the entire protein-protein complex (SoxYZCD) were shown in the left hand side. On the right hand side, the interacting residues and the hydrogen bonds formed upon interaction with thiosulphate was also depicted. Furthermore, for the analysis of steady interaction after simulation, the electrostatic surface potentials upon SoxYZ protein complexes and conformational fluctuations in SoxY as well as SoxZ protein upon SoxCD interaction was illustrated through figures. The table at the end shows the steady and spontaneous interaction within the complex (through the calculation of statistically significant free energy of folding value and net area for solvent accessibility for the interacting residues). [file 8683713.f1.doc]

Supplementary Materials Legends

| **Supplementary Material Number** | **Supplementary Material Caption** |
| --- | --- |
| **Suppl. Fig. 1** | Overall study for the entire work flow in a single pictorial representation |

**Supplementary Material**

**
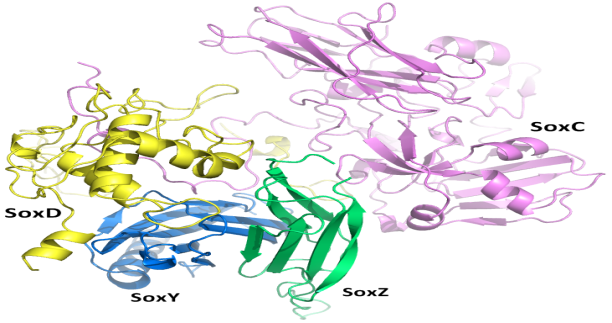
**
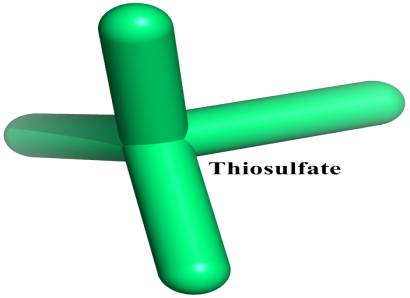
**
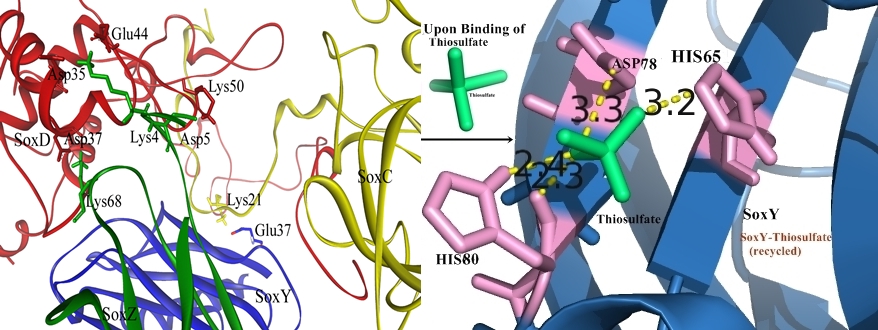

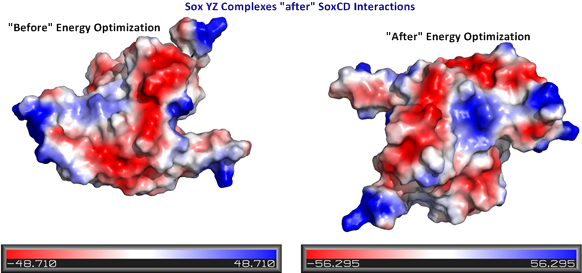
**
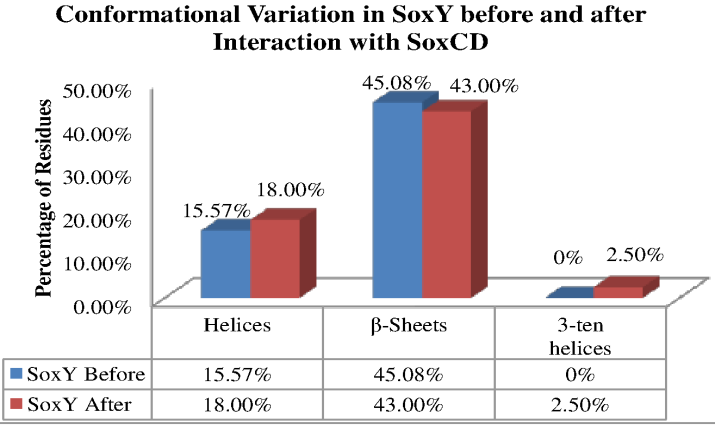

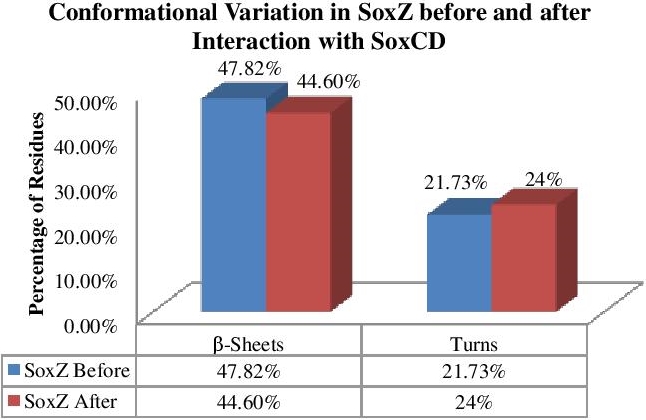


| **Stability Parameters** | **Before Interaction** | **After Interaction** |
| --- | --- | --- |
| Free energy of folding (kcal/mol) | -144.71 | -577.31 |
| Net area for solvent accessibility of  interacting residues | 486.92Å^2^ | 153.92Å^2^ |
|  |  |  |

**Suppl. Fig. 1** Overall study for the entire work flow in a single pictorial representation
